# Bioconversion of Styrene to Poly(hydroxyalkanoate) (PHA) by the New Bacterial Strain *Pseudomonas putida* NBUS12

**DOI:** 10.1264/jsme2.ME14138

**Published:** 2015-02-14

**Authors:** Giin-Yu Amy Tan, Chia-Lung Chen, Liya Ge, Ling Li, Swee Ngin Tan, Jing-Yuan Wang

**Affiliations:** 1 Residues and Resource Reclamation Centre, Nanyang Environment and Water Research Institute, Nanyang Technological University1 Cleantech Loop, Singapore 637141Singapore; 2 Division of Environmental and Water Resources Engineering, School of Civil and Environmental Engineering, Nanyang Technological University50 Nanyang Avenue, Singapore 639798Singapore; 3 Natural Sciences and Science Education Academic Group, Nanyang Technological University1 Nanyang Walk, 637616Singapore

**Keywords:** styrene, eco-pollutants, medium-chain-length poly(hydroxyalkanoate) (mcl-PHA), biopolyester, *Pseudomonas putida* NBUS12

## Abstract

Styrene is a toxic pollutant commonly found in waste effluents from plastic processing industries. We herein identified and characterized microorganisms for bioconversion of the organic eco-pollutant styrene into a valuable biopolymer medium-chain-length poly(hydroxyalkanoate) (mcl-PHA). Twelve newly-isolated styrene-degrading Pseudomonads were obtained and partial *phaC* genes were detected by PCR in these isolates. These isolates assimilated styrene to produce mcl-PHA, forming PHA contents between 0.05±0.00 and 23.10±3.25% cell dry mass (% CDM). The best-performing isolate was identified as *Pseudomonas putida* NBUS12. A genetic analysis of 16S rDNA and *phaZ* genes revealed *P. putida* NBUS12 as a genetically-distinct strain from existing phenotypically-similar bacterial strains. This bacterium achieved a final biomass of 1.28±0.10 g L^−1^ and PHA content of 32.49±2.40% CDM. The extracted polymer was mainly comprised of 3-hydroxyhexanoate (C_6_ ), 3-hydroxyoctanoate (C_8_ ), 3-hydroxydecanoate (C_10_ ), 3-hydroxydodecanoate (C_12_ ), and 3-hydroxytetradecanoate (C_14_ ) monomers at a ratio of 2:42:1257:17:1. These results collectively suggested that *P. putida* NBUS12 is a promising candidate for the biotechnological conversion of styrene into mcl-PHA.

Styrene is a very important industrial mono-aromatic compound that is primarily used in the production of petrochemical-based plastics such as poly(styrene) (PS), acrylonitrile butadiene styrene (ABS), styrene acrylonitrile (SAN), and poly(ethylene) (PE). Singapore, as one of the world’s major petrochemical and refining hubs, is also a key producer of styrene monomers and styrene-derived products, accounting for over 1% of global production (2). Waste effluents generated from plastic production plants are often rich in unreacted styrene; concentrations may be approximately 15,000-fold higher than that recommended by the WHO guidelines (1, 48). Styrene is also present in effluents from chemical, textile latex, and coal gasification plants (1). In addition to industrial effluents, solid waste from post-consumer styrene-derived plastic products also pose as an environment burden due to their non-biodegradability, leading to their accumulation in landfills and dump sites, while inappropriately-discarded plastics ultimately become a principal component of marine debris (6). Styrene pollution is associated with negative ecological impacts (5), and serious health effects including depression of the central nervous system, damage to the liver, potential endocrine disruptions, and cancer (48). Therefore, the treatment of styrene-laden effluents and styrene-based solid wastes is pivotal for environment and public health protection, and an issue of both local and international importance.

Bioconversion is an attractive option for treating styrene as it uses microorganisms as biocatalysts for the transformation of mono-aromatic contaminants into organic biomass and harmless compounds such as carbon dioxide and water. Significant efforts have been made to isolate styrene-degrading bacterial strains, such as members from *Pseudomonas*, *Rhodococcus*, *Bacillus*, and *Xanthobacter* (41), and apply them to the bioremediation of styrene-contaminated effluents, off-gas, and soils (7, 8, 12).

Bioremediation potentially allows for the bioconversion of waste into valued products such as poly(hydroxyalkanoate) (PHA) (14, 40). PHA has attracted commercial interest due to its chemically diverse, biodegradable, and biocompatible properties, giving rise to its various applications ranging from biodegradable packaging materials to pharmaceutical products (34, 50). In spite of the wide application potentials of PHAs, most, particularly medium-chain length PHA (mcl- PHA), have yet to be commercialized due to their high production costs. One effective way to reduce production costs is the use of less expensive carbon substrates (3, 4, 23, 24, 31). The pyrolysis of styrene-based plastic waste, particularly PS, can produce pyrolytic oil that comprises up to 75% styrene (22). Hence, pyrolytic oil from plastic waste and industrial styrene effluent present an alternative source of cheap or freely-available carbon substrates for the production of PHA (46). However, few efforts have been made to isolate and characterize bacterial strains that are able to assimilate styrene for the production of mcl-PHA. Only four styrene-degrading bacterial strains: *Pseudomonas* sp. TN301, *P. putida* CA-3 (NCIMB 41162), *P. putida* CA-1, and *P. putida* S12, have so far been shown to be capable of mcl-PHA accumulation (25, 42, 45). However, most of these bacteria accumulate PHA at low cellular contents of between 3 and 14% cell dry mass (% CDM). The only exception is *P. putida* CA-3, a patent strain that can store mcl-PHA up to 33% CDM and 31.8% CDM under shake-flask and fermenter conditions, respectively (27, 28). Therefore, the pool of bacterial strains with the metabolic capacity to efficiently bioconvert styrene into mcl-PHA needs to be increased. The aim of the present study was to isolate and characterize new styrene-degrading *cum* PHA-producing bacterial strains as a means to facilitate the development of a biotechnology that not only provides an environmentally friendly treatment approach to address the issue of styrene-based aqueous/solid waste, but also has the potential benefits of lowering PHA production costs and off-setting biological treatment costs through the recovery of PHA.

## Materials and Methods

### Isolation of styrene-degrading bacteria

Pure bacterial cultures were isolated from two aerobic bioreactors that were seeded with domestic activated sludge and industrial activated sludge from a local water reclamation plant and petrochemical wastewater treatment facility, respectively. The sludge inoculums were enriched on 1× mineral salt medium (MSM), containing 3.70 g L^−1^ KH_2_ PO_4_ , 5.80 g L^−1^ K_2_ HPO_4_ , 0.2 g L^−1^ MgSO_4_ .7H_2_ O, and 1.0 mL L^−1^ microelement solution (2.78 g L^−1^ FeSO_4_ .7H_2_ O, 1.98 g L^−1^ MnCl_2_ .4H_2_ O, 2.81 g L^−1^ CoSO_4_ .7H_2_ O, 1.67 g L^−1^ CaCl_2_ .2H_2_ O, 0.17 g L^−1^ CuCl_2_ .2H_2_ O, and 0.29 g L^−1^ ZnSO_4_ .7H_2_ O in 0.1 M HCl). The pH of MSM was adjusted to 7.2 using 3 M NaOH. The nitrogen source used in this study was 16 mM of (NH_4_ )_2_ SO_4_ unless otherwise stated. A mono-aromatic mixture, comprising styrene, benzene, toluene, ethylbenzene, and *p*-xylene (BTEX) at an equimolar concentration of 1.8 mM, was provided as the sole carbon source. The cultivation media in the bioreactors were replaced with fresh media weekly. After 11 weeks of enrichment, bacterial isolation was performed on 1× MSM agar containing 1 g L^−1^ sodium benzoate. The sludge samples from the two bioreactors were serially diluted (10^−1^ to 10^−4^) in 1× phosphate buffer saline (PBS) and 100 μL of each dilution was evenly spread on the agar plates. The plates were incubated at 30°C for 2 to 5 days. The colonies that appeared on the agar were transferred onto fresh agar until pure bacterial isolates, as determined by colony morphology and color, were obtained. Bacterial isolates, obtained from domestic activated sludge, were denoted with the prefix “NBUS” while those obtained from industrial activated sludge were denoted with the prefix “NBIWW”.

To test the styrene-utilization capability of the pure bacterial isolates, a bacterial starter culture for each isolate was cultivated with 1× MSM containing 1 g L^−1^ of sodium benzoate with shaking at 100 rpm, 30°C for 2 days. The bacterial culture (10 μL) was pipetted onto 1× MSM agar containing 40 μL styrene supplied by placing a pipette tip on the agar. The petri dish was tightly sealed with para-film and placed in an air-tight jar at 30°C for up to 5 days. A negative control setup, without carbon substrate, was included.

### 16S rDNA and *phaC* genetic characterization of styrene-degrading bacterial isolates

The total DNA of styrene-degrading bacterial isolates was extracted using a chemical-lysis method described by Liu *et al.* (18). Extracted DNA material served as a template for the PCR amplification of bacterial 16S rDNA using the primer set 8F/1490R (47) and PHA synthase *phaC* gene using the general primer set G-D/G-1R, which amplified a section of the genes encoding Class I PHA synthase (*phaC*) and Class II PHA synthases (*phaC1* and *phaC2*) (35). The PCR reaction mixture consisted of 1× GoTaq Colorless Mastermix (Promega, Madison, WI, USA), with 0.2 μM of each primer, 3.0 mM MgCl_2_ , 1 μL of DNA template, and nuclease-free water to complete the 50-μL reaction mixture. Amplification was performed on a Bio-Rad C1000 Thermal Cycler (Bio-Rad Laboratories, Hercules, CA, USA) according to temperature programs previously described (35, 47). Sequencing of the 16S rDNA PCR product was performed by a local sequencing service company (Axil Scientific, Singapore). The partial 16S rDNA sequences of the bacterial isolates (>1,400 bp) were compared to available sequences in GenBank using the NCBI’s BLAST program. MEGA5.2 software (38) was used to align the 16S rDNA sequences (with provided ClustalW function) and construct a neighbor-joining tree with the Juke-Cantor correction. Bootstrapping for 1,000 replicates was used to estimate the confidence of the tree’s topology. Partial 16S rDNA sequence data were deposited to GenBank under accession numbers KF765785 to KF765796.

### Screening for PHA accumulation in styrene-degrading bacterial isolates

Bacterial starter cultures were transferred to a 250-mL conical flask, containing 50 mL of 1× MSM and 0.25 g L^−1^ NH_4_ Cl (67 mg L^−1^ nitrogen; nitrogen-limited), at an initial optical density at 600 nm (OD_600_ ) of 0.01. Styrene (350 μL) was added to a centrally fused column, which enabled styrene to partition into the headspace and liquid medium. The conical flasks were tightly sealed and incubated at 30°C, 200 rpm. After 48 h, the bacterial cultures were centrifuged (9,840×*g*, 15°C, 10 min) to harvest cell pellets. Cell pellets were washed twice with 1× PBS buffer and freeze-dried. The cell dry mass (CDM) of the freeze-dried biomass was determined. The PHA content was quantified by subjecting dried biomass (5 to 10 mg) to methanolysis followed by a GC-MS analysis, at a split ratio of 50:1, according to the procedures described by Tan *et al.* (39).

### PCR amplification of the *phaZ* gene and phylogenetic tree construction

In order to amplify the PHA depolymerase *phaZ* gene, the primers DEV15R and DEV15L (36), were reverse-complemented and modified to DEV15R-RC (5′-GCA TCG GCG CCA ACC TGG-3′) and DEV15L-RC (5′-GRA ACT TCA TGA TGA TCG GGG-3′). Amplification was performed with the following temperature program: 1 cycle at 95°C for 5 min, followed by 25 cycles at 94°C for 30 s, 63.4°C for 30 s and 72°C for 45 s, and a final cycle at 72°C for 5 min. The DNA template from the known PHA-accumulator *P. putida* mt-2 (NCIMB 10432) served as a positive control while that from the non-PHA accumulator *Escherichia coli* CN13 (ATCC 700609) served as a negative control. A PCR reaction without the DNA template was also included to serve as an empty control. Sequencing of the *phaZ* PCR product was performed by Axil Scientific (Singapore). Partial *phaZ* sequences (>600 bp) were compared to available sequences in GenBank using the NCBI’s BLAST program and also to other existing PHA-producing Pseudomonads described by Solaiman and Ashby (36). Phylogenetic tree construction, based on partial *phaZ* sequences, was performed using MEGA5.2 software (38) according to previously described procedures. The phylogenetic tree of PhaZ protein sequences was constructed using the neighbor-joining (*p*-distance) algorithm with bootstrapping for 1,000 replicates to estimate the confidence of the tree’s topology. The partial *phaZ* sequences were submitted to GenBank under accession numbers KF765798 (*P. putida* NBUS12) and KF765797 (*P. putida* mt-2). The partial PhaZ sequences were assigned protein ID numbers AHG53077 (*P. putida* NBUS12) and AHG53076 (*P. putida* mt-2).

### Biochemical assay, temperature, and pH shake-flask studies

Gram staining was performed with a three-step Gram stain procedure kit (Becton Dickinson Microbiology Systems, Cockeysville, MD, USA). A biochemical assay was performed using Biolog GEN III MicroPlate™ and analyzed using Biolog MicroLog 3 v5.2.01 software (Biolog, Hayward, CA, USA). In the temperature and pH experiments, the bacterial starter culture was inoculated to 10 mL of 1× MSM, containing 1 g L^−1^ of sodium benzoate, at an initial OD_600_ of 0.05. To determine the optimum growth temperature of the selected bacterial isolate, the bacterial culture was incubated at four different temperatures (*i.e.*, 25, 30, 35, and 40°C). The optimum pH for the growth medium was investigated at 30°C in the pH range of 3 to 11 (*i.e.*, 3, 4, 5.5, 6, 7, 8, 10, and 11) with pH adjustments by the addition of either HCl or NaOH. Bacterial growth over the course of 72 h was monitored by changes in OD_600_ readings with a Cary UV-Vis spectrophotometer (Agilent Technologies, Palo Alto, CA, USA) at 12-h and 24-h intervals in the temperature and pH experiments, respectively.

### Transmission electron microscopy (TEM)

Bacterial cells were recovered by centrifugation (9,425×*g*, 10 min). Cell pellets were resuspended in fixation buffer, containing 2% (v/v) paraformaldehyde and 2.5% (v/v) glutaraldehyde, for 2 h. Following fixation, the immobilized cells were washed thrice with 0.5× PBS and post-fixed with 1% (v/v) osmium tetraoxide for 2 h. The cells were washed thrice with 0.5× PBS and dehydrated through a graded acetone series. The dehydrated cells were embedded with propylene oxide and Spurr resin (1:1 ratio [v/v]) and polymerized. Ultrathin sections, between 70 and 100 nm in thickness, were cut with a Leica UCT ultramicrotome (Leica Microsystems, Bannockburn, IL, USA). Ultrathin sections were stained with uranyl acetate followed by lead citrate before viewing with JEOL JEM 2010F TEM (JEOL, Tokyo, Japan), equipped with a field-emission gun, with an accelerating voltage of 200 kV. Imaging was performed at magnifications of 25,000 to 40,000×. Image capture was performed with a GATAN charge-coupled device camera and analyzed using commercial GATAN software (Gatan, Pleasanton, CA, USA).

### Bacterial cultivation for PHA polymer extraction

An overnight bacterial starter culture was inoculated into a 1-L cultivation reactor containing 500 mL of 1× MSM at an initial OD_600_ of 0.1. Temperature and agitation were controlled at 30°C and 100 rpm, respectively. The initial pH of the cultivation media was adjusted to 7.19±0.15. A styrene reservoir was set up using a serum bottle and placed in between the air pump and cultivation reactor. The air pump was connected to the inlet of the reservoir to induce the volatilization of styrene. Gaseous styrene exited the reservoir via a reservoir outlet and was supplied to the cultivation reactor via an air sparger. The flow rate of the gas mixture was set to 500 mL min^−1^ using an airflow regulator, delivering gaseous styrene to the reactor at an average rate of 3.63±0.54 g h^−1^. The nitrogen source NH_4_ Cl was added at an initial concentration of 191 mg L^−1^ (50 mg L^−1^ nitrogen) with further additions of 95.5 mg L^−1^ (25 mg L^−1^ nitrogen) at 24 and 48 h to maintain nitrogen at a limiting level for PHA accumulation. After 72 to 120 h of cultivation, cell pellets were harvested from bacterial cultures by centrifugation (9,840×*g*, 15°C, 10 min) and washed twice with 1× PBS prior to freeze-drying. The PHA polymer from 4.58 g freeze-dried cells was extracted with dichloromethane (10% [w/v]) at 55°C for 8 h using a Soxhlet apparatus. The extract was filtered through Whatman No.4 filter paper to remove cellular debris. Ice-cold methanol was slowly added to the filtrate at 1:1 (v/v) ratio under vigorous stirring to precipitate PHA. PHA was subsequently separated from the methanol-dichloromethane mixture by centrifugation at 9,425×*g* (−1°C, 5 min), casted on a glass surface, and air-dried overnight.

### PHA polymer analysis

The PHA polymer (5 to 10 mg) was derivatized according to the procedures described by Tan *et al.* (39) for GC-MS analysis. In the ^1^H-NMR and ^13^C-NMR analyses, 30 mg of the PHA polymer was dissolved in 0.7 mL deuterated chloroform containing 0.03% (v/v) tetramethylsilane (TMS). The solution was transferred to a NMR tube (5 mm O.D., 7 inch length; Sigma-Aldrich, St Louis, MO, USA). NMR spectra were recorded on a Bruker DRX-400 spectrometer at 400 MHz (Bruker, Switzerland) using deuterated chloroform as a solvent and TMS as an internal standard. Chemical shifts were given in ppm (δ) relative to the chemical shift of solvent residual signals for deuterated chloroform at 7.26 and 77.00 ppm, respectively.

The weight average molar mass (*M_w_*), number average molar mass (*M_n_*), and polydispersity index (PDI) were determined by gel permeation chromatography (GPC). An Agilent 1100 series GPC system equipped with a LC pump, PLgel MIXED-C column (5 μm, 7.5×300 mm), and refractive index (RI) detector were used (Agilent Technologies). The column was calibrated with polystyrene standards (EasiVial PS-H; Agilent Technologies). Tetrahydrofuran (THF), containing 250 ppm of 2,6-di-tert-butyl-4-methylphenol (BHT) as an inhibitor, was used as the mobile phase at a flow rate of 1 mL min^−1^.

The glass transition temperature (*T_g_*) and melting temperature (*T_m_*) of the PHA polymer were determined using differential scanning calorimetry (DSC) on Mettler-Toledo DSC1 (Mettler-Toledo, Switzerland) at a heating rate of 10°C min^−1^ from −70 to 80°C with a nitrogen purge at 80 mL min^−1^. The mid-point of the heat capacity change was determined as *T_g_* while the maximum endothermic point was taken as *T_m_*. The decomposition temperature (DT) was determined as the onset temperature of the derivative thermogravimetry curve using a thermogravimetric analysis (TGA) on Netzsch STA 499 F3 (Netzsch, Germany) with a heating rate of 10°C min^−1^ from 35°C to 600°C under a nitrogen flow rate of 50 mL min^−1^. The crystallinity of the PHA polymer was determined by powder X-ray diffraction (XRD) (D8 Advance; Bruker AXS, Karlsruhe, Germany) using Cu-Kα radiation (Kα=1.5418 Å) over a 2θ range of 10 to 50°, 0.03° min^−1^, 1 s step^−1^.

## Results

### Styrene-degrading bacterial isolation and identification

**Fig. 1:**
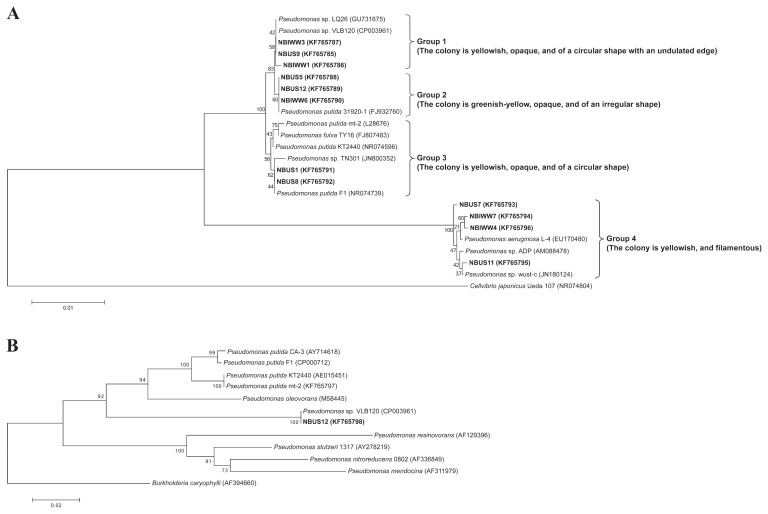
Neighbor-joining phylogenetic tree based on (A) the partial 16S rDNA sequences of bacterial isolates (designated in bold with a description of the colony morphology on styrene agar medium) and closely-related bacteria with *Cellvibrio japonicas* Ueda107 (NR074804) as the outgroup, and (B) the partial *phaZ* gene sequence of *P. putida* NBUS12 (designated in bold), *Pseudomonas* sp. VLB120, and known PHA-producing Pseudomonad strains described by Solaiman and Ashby (36) with *Burkholderia caryophylli* as the outgroup. GenBank accession numbers are provided within parentheses. The scale bar represents the estimated number of nucleotide changes per sequence position.

Sodium benzoate was used as the aromatic carbon source for isolation due to its solubility, non-volatility, and non-toxic nature. Previous studies demonstrated that sodium benzoate-utilizing bacterial strains were also capable of degrading styrene (17). A total of 16 bacterial isolates were obtained on agar medium containing sodium benzoate. Of these, 12 isolates were able to grow using styrene as the sole carbon source. Seven bacterial isolates were obtained from domestic activated sludge while 5 bacterial isolates were obtained from industrial activated sludge. The 16S rDNA sequences of the styrene-utilizing bacterial isolates revealed that all the bacterial isolates had a close evolutionary relationship with the genus *Pseudomonas*, which is a family of bacteria known for its mono-aromatic biodegradation properties including styrene metabolism (11). Four distinct groups were identified based on phylogeny analysis and colony morphology (Fig. 1A). These groups were Group 1 (NBUS9, NBIWW1, and NBIWW3), Group 2 (NBUS5, NBUS12, and NBIWW6), Group 3 (NBUS1 and NBUS8), and Group 4 (NBUS7, NBUS11, NBIWW4, and NBIWW7). With the exception of Group 3, which comprised of bacterial isolates from domestic activated sludge, the other three groups consisted of isolates from both domestic and industrial activated sludge.

The phylogenetic analysis revealed that Group 1 and 2 bacterial isolates were closely related to the styrene degraders *Pseudomonas* sp. LQ26 and *Pseudomonas* sp. VLB120 (16, 30) while Group 4 bacterial isolates were closely related to the atrazine-degrader *Pseudomonas* sp. ADP (21) (Fig. 1A). Although *Pseudomonas* sp. LQ26, *Pseudomonas* sp. VLB120, and *Pseudomonas* sp. ADP are capable of degrading aromatic compounds, they are not known to accumulate PHA. On the other hand, Group 3 bacterial isolates were clustered together with *Pseudomonas* strains known to accumulate PHA from mono-aromatics such as styrene, BTEX, and/or non-aromatic substrates. These strains included *P. fulva* TY16, *P. putida* F1, *P. putida* mt-2, *Pseudomonas* sp. TN301, and *P. putida* KT2440 (25, 26, 27, 44). With the exception of *Pseudomonas* sp. TN301, the 16S rDNA gene sequences of most of the existing styrene-degrading and PHA-producing *Pseudomonas* strains (*i.e.*, *P. putida* CA-3, *P. putida* CA-1, and *P. putida* S12) were unavailable. Hence, genetic comparisons were made between the four groups of styrene-utilizing bacterial isolates from this study and *Pseudomonas* sp. TN301. Group 1, Group 2, and Group 3 bacterial isolates showed 99% identity with 97% coverage with *Pseudomonas* sp. TN301, while Group 4 bacterial isolates showed 96% identity with 96% coverage with *Pseudomonas* sp. TN301.

### Bioconversion of styrene to PHA by bacterial isolates

PHA synthesis in Pseudomonads is generally initiated under nitrogen-limiting conditions in the presence of excess carbon (45), and is executed by two Class II PHA synthase genes (*phaC1* and *phaC2*). The PHA synthase genes flank a PHA depolymerase gene (*phaZ*), which is responsible for PHA degradation (36). Using a general primer set for the PCR detection of Class I and Class II PHA synthase genes, a PCR product with an expected size of approximately 500 bp was obtained for all bacterial isolates (data not shown), indicating the presence of *phaC* genes and, therefore, the genetic potential of the bacterial isolates to synthesize PHA.

The four groups of bacterial isolates were tested for their ability to bioconvert styrene into PHA. Under the nitrogen-limiting conditions in the shake-flask culture, Group 1, 3, and 4 bacterial isolates attained similar final CDM between 0.26±0.04 g L^−1^ and 0.37±0.19 g L^−1^ (Table 1). In terms of PHA content, Group 1 and 4 bacterial isolates accumulated less than 0.1% CDM of mcl-PHA, consisting of 3- hydroxydecanoate (C_10_ ) and 3-hydroxydodecanoate (C_12_ ) monomers in a C_10_ :C_12_ ratio of 82:18 and 52:48, respectively, and had low PHA mass fractions (Group 1, 0.55±0.03 mg gCDM^−1^; Group 4, 0.79±0.13 mg gCDM^−1^). Group 3 bacterial isolates had a higher mcl-PHA content and PHA mass fraction of 6.23±1.46% CDM and 62.25±14.62 mg gCDM^−1^, respectively (Table 1). Mcl-PHA produced by Group 3 bacterial isolates comprised 3-hydroxyhexanoate (C_6_ ), 3-hydroxyoctanoate (C_8_ ), C_10_ , and C_12_ monomers at a C_6_ :C_8_ :C_10_ :C_12_ ratio of 4:30:64:2 with C_10_ and C_8_ monomers predominating the polymer (94 weight % [wt %]). Among the four groups, the highest growth and PHA formation were observed in Group 2 bacterial isolates (0.63±0.14 g L^−1^; 13.67±5.22% CDM) (Table 1). Similar to Group 3 bacterial isolates, the monomeric composition of mcl-PHA was mostly comprised of C_8_ and C_10_ monomers (96 wt %) with a C_6_ :C_8_ :C_10_ :C_12_ ratio of 1:20:76:3. Within Group 2, the bacterial isolate NBUS12 showed the highest growth on styrene and PHA content, and was selected for further investigation.

### Biochemical and genetic characterization of the bacterial isolate NBUS12

*Pseudomonas* isolate NBUS12 is an aerobic Gram-negative rod-shaped bacterium, approximately 1.5 μm in size, and was identified as the species “*putida*” by the Microlog database. This bacterium can grow at temperatures between 25 and 35°C, and pH values ranging from 6 to 11 (data not shown). A Biolog assay also revealed that the bacterium had high reducing power and tolerated growth conditions up to 4% NaCl. Table 2 provides a summary of the various carbon sources on Biolog GEN III MicroPlate™ that *P. putida* NBUS12 was able to assimilate, as well as the antibiotic resistance of *P. putida* NBUS12. Under shake-flask conditions, *P. putida* NBUS12 grew to a final biomass of 0.80±0.03 g L^−1^, comprising of PHA at 23.10±3.25% CDM (Table 1). The C_6_ :C_8_ :C_10_ :C_12_ monomeric composition was 1:15:82:2 with C_8_ and C_10_ monomers accounting for the bulk of the polymer at 97 wt % (Table 1).

Genetic comparisons were made between *P. putida* NBUS12 and *Pseudomonas* sp. TN301 using 16S rDNA sequences, and between *P. putida* NBUS12, *P. putida* CA-3 and other known PHA-producing Pseudomonads (36) using *phaZ* sequences. The 16S rDNA sequence of *P. putida* NBUS12 shared 99% identity with 97% coverage with *Pseudomonas* sp. TN301, but showed higher genetic similarity to *P. putida* 31920-1 (100% identity, 99% coverage), which is a bacterium strain not known to accumulate PHA (Fig. 1A). The *phaZ* gene of *P. putida* NBUS12 also did not show particularly high genetic similarity to *P. putida* CA-3 (88% identity with 100% coverage) and other known PHA-producing Pseudomonads (80 to 88% identities with 99 to 100% coverage). A BLAST analysis identified *Pseudomonas* sp. VLB120 as *P. putida* NBUS12’s closest relative (100% identity with 100% coverage). A ClustalW analysis of *phaZ* sequences indicated that *P. putida* NBUS12 was evolutionarily closer to PHA-producing *P. putida* than other PHA-producing *Pseudomonas* species (*i.e.*, *P. resinovorans*, *P. stutzeri*, *P. nitroreducens*, and *P. mendocina*), but still formed a distinct branch together with *Pseudomonas* sp. VLB120, which is a styrene-degrader not known to produce PHA (Fig. 1B). Further analyses with *phaZ* translated amino acid sequences showed that *P. putida* NBUS12 had 100% coverage with 98% and 100% identities to *P. putida* CA-3 and *Pseudomonas* sp. VLB120, respectively. Phylogenetic clustering of the PhaZ sequences revealed similar results to those for *phaZ* sequences; *P. putida* NBUS12 was grouped together with *Pseudomonas* sp. VLB120 as an evolutionarily distinct branch (Fig. S1).

### Characterization of the PHA polymer produced from styrene by *P. putida* NBUS12

**Fig. 2:**
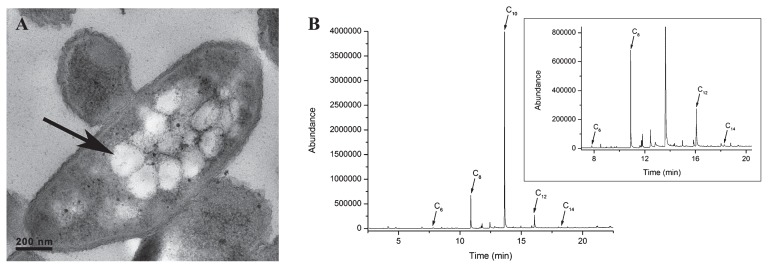
(A) TEM micrograph of a *P. putida* NBUS12 bacterium with intracellular mcl-PHA granules. The arrow indicates a mcl-PHA granule. (B) GC-MS chromatogram of mcl-PHA monomers detected in the polymer extracted from *P. putida* NBUS12. The insert shows a magnified version of the chromatogram in which the C_10_ peak had been truncated (C_6_ , 3-hydroxyhexanoate; C_8_ , 3-hydroxyoctanoate; C_10_ , 3-hydroxydecanoate; C_12_ , 3-hydroxydodecanoate; C_14_ , 3-hydroxytetradecanoate).

To obtain sufficient biomass for mcl-PHA polymer extraction, the cultivation volume of *P. putida* NBUS12 was increased to 500 mL. Each cultivation run produced an average of 1.28±0.10 g L^−1^ biomass with 32.49±2.40% CDM of PHA content, corresponding to a total PHA production of 0.42±0.04 g L^−1^ and PHA mass fraction of 324.90±23.98 mg gCDM^−1^. Under TEM analysis, the bioaccumulated mcl-PHA was visible as intracellular PHA granules within *P. putida* NBUS12 (Fig. 2A). A GC-MS analysis of the methanolyzed polymer revealed the presence of medium-chain-length C_6_ , C_8_ , C_10_ , C_12_ monomers, and an additional 3-hydroxytetradecanoate (C_14_ ) monomer with retention times of 7.82, 10.89, 13.64, 16.07, and 18.26 min, respectively (Fig. 2B). The retention time profile was consistent with previous findings (39). The monomers were present at a C_6_ :C_8_ :C_10_ :C_12_ :C_14_ ratio of 2:42:1257:17:1 with the C_10_ monomer accounting for 95 wt % of the polymer.

**Fig. 3:**
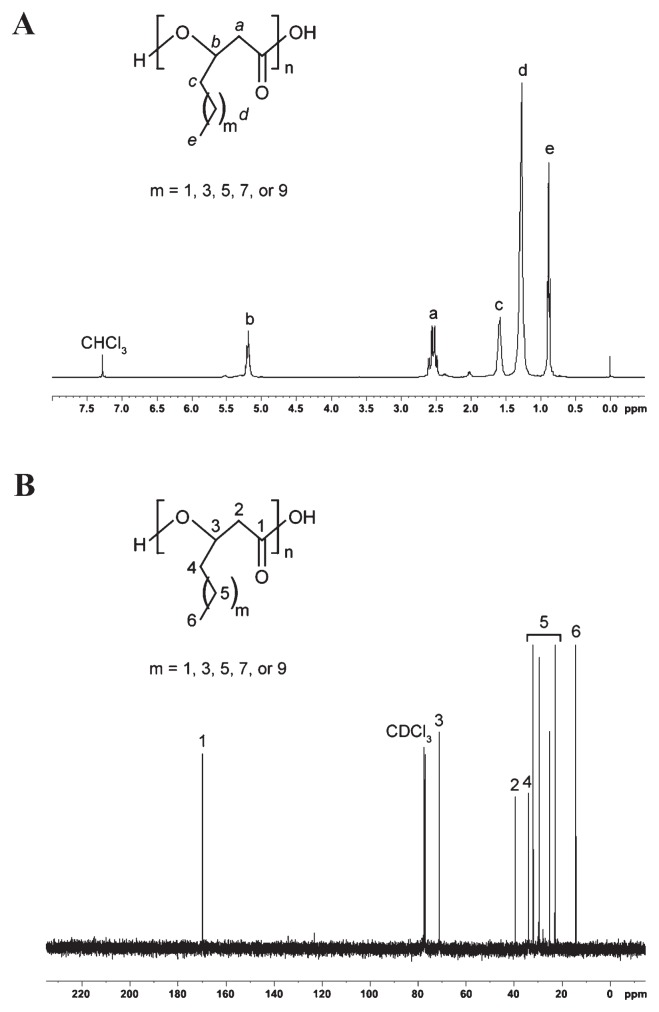
(A) ^1^H-NMR and (B) ^13^C-NMR spectra of the mcl-PHA polymer from *P. putida* NBUS12 using styrene as the sole carbon substrate. The signals corresponding to hydrogen atoms in the ^1^H-NMR spectrum are denoted by letters while the signals corresponding to carbon atoms in ^13^C-NMR spectrum are denoted by numbers.

The NMR analytical results of the PHA polymer are shown in Fig. 3. Collectively, GC-MS and NMR analyses suggested that *m* may have a value of 1, 3, 5, 7, and 9, corresponding to the C_6_ , C_8_ , C_10_ , C_12_ , and C_14_ monomeric units, respectively. The NMR chemical shifts of different PHA monomeric units are compiled in Table S1, which were in good agreement with previous findings and displayed the typical profiles of mcl-PHA co-polymers (9, 10, 24). The additional signals at 2.02, 2.35, 5.29, and 5.53 ppm in the ^1^H-NMR spectrum and signals between 123.3 ppm and 134.2 ppm in the ^13^C-NMR spectrum indicated the presence of olefinic groups along the alkyl side chain. Therefore, besides saturated monomers, low levels of unsaturated monomers were also present in the polymer. The percentage of unsaturated units in PHA was estimated from the ratio of the integration peak at 2.02 ppm to peak at 2.52 ppm in the ^1^H-NMR spectrum. The PHA produced by the new bacterial isolate *P. putida* NBUS12 contained an abundance of approximately 7.4% of unsaturated units. However, due to the lack of analytical standards, the chemical structures of these unsaturated monomers have yet to be elucidated and further verified.

The *M_w_* and *M_n_* of the mcl-PHA polymer were 101,500 Da and 49,300 Da, respectively, while the PDI index was 2.06 (Table 3). *T_g_* was −46.05°C and *T_m_* was 50.67°C, giving rise to the amorphous properties of the polymer at room temperature. The polymer was also partially crystalline (13.57%) with crystalline peaks being detected at 2θ values of 19.05 and 21.68 under the XRD analysis. Thermal degradation of the polymer occurred at 277°C.

## Discussion

Environmental pollution with styrene and styrene-derived products is pervasive in land, water and air, particularly in effluents and off-gases from petrochemical industries (1, 41). Styrene-based waste presents a large volume of an inexpensive carbon source that can be bioconverted and upcycled to a high-value mcl-PHA biopolymer, potentially lowering the price of PHA and off-setting waste treatment costs. While styrene bioremediation has been examined extensively, the bioconversion of styrene into a new resource such as mcl-PHA has received limited attention. This study aimed at addressing this research gap through the isolation and characterization of bacteria that may be able to bioconvert styrene into mcl-PHA.

Aromatic-degrading and PHA-forming bacteria are generally isolated from petrochemical-contaminated sites or bioreactors treating petrochemical industrial effluents (13, 25, 26) because these pre-enriched sources are more likely to yield microorganisms that could break down petrochemical aromatics. In the present study, activated sludge from a water reclamation plant treating municipal wastewater and industrial activated sludge from a petrochemical wastewater treatment facility were tested as inoculum sources. Following 11 weeks of enrichment on a mono-aromatic mixture containing styrene as the sole carbon source, 16 benzoate-degrading bacterial isolates were isolated. Four bacterial isolates could not grow on styrene whereas 12 bacterial isolates were found to be capable of consuming styrene. Benzoate is one of the by-products of styrene degradation (41) and the isolation of a minority of bacteria, which were able to consume benzoate, but not styrene, suggested the emergence of a dominant styrene-degrading microbial population supporting a sub-microbial population that thrives on styrene degradation intermediates. Based on 16S rDNA sequences, the 12 bacterial isolates were all identified as Pseudomonads and further classified into four distinct groups with most groups comprising of bacterial isolates from both domestic and industrial activated sludge (Fig. 1A). The *Pseudomonas* genus is a family of bacteria with well-known aromatic bioremediation and mcl-PHA accumulation properties (40). BLAST and phylogeny analyses revealed that the bacterial isolates had a close evolutionary relationship with known aromatic-degrading and/or mcl-PHA-producing *Pseudomonas* strains including *Pseudomonas* sp. LQ26, *Pseudomonas* sp. VLB120, *Pseudomonas* sp. ADP, *P. fulva* TY16, *P. putida* F1, *P. putida* mt-2, *Pseudomonas* sp. TN301, and *P. putida* KT2440 (16, 21, 25, 26, 27, 30, 44) (Fig. 1A). All the bacterial isolates were from the *Pseudomonas* genus, and the pre-enriched nature of industrial activated sludge did not result in any marked difference in the type of bacterial isolates obtained when compared to domestic activated sludge. A possible explanation may be that Pseudomonads are *r*-strategists, which are highly adaptive to nutrient-rich, unstable, and uncrowded environments, enabling them to rapidly proliferate and dominate the microbial population (15). The *r*-strategy growth of Pseudomonads, coupled with their broad aromatic catabolic properties (19), may have provided them with a competitive edge to thrive under the strong selective pressure induced by recalcitrant monoaromatic substrate feed, forming a more dominant population in the sludge microbial community during the early stages of enrichment and at the point of this study’s sampling.

Partial *phaC* genes were detected in all four groups of bacterial isolates and, under nitrogen-limiting shake-flask conditions, these isolates bioconverted styrene into mcl-PHA (Table 1). Hence, within the context of this study, it appears that a short enrichment period (*i.e.*, 11 weeks) for a non-contaminated inoculum source, such as domestic activated sludge, was sufficient to yield styrene-degrading bacteria with mcl-PHA accumulation properties, providing a relatively simple and fast enrichment strategy. The mcl-PHA detected in all groups comprised C_6_ , C_8_ , C_10_ , and C_12_ monomers (Table 1), which was consistent with previous findings (25, 27). Previous studies suggested that the bioconversion process may begin with the side-chain oxidation of styrene and *β*-oxidation to form acetyl-CoA through phenylacetaldehyde before acetyl-CoA enters the downstream *de novo* fatty acid synthesis pathway that generates PHA precursors of varying chain lengths for PHA polymerization (29). Aside from the aforementioned styrene catabolism pathway, aerobic styrene degradation is also known to occur via side-chain oxidation to form benzoate intermediates and also through direct ring cleavage (41). While there is currently no evidence of coupling between these two styrene catabolism pathways and the mcl-PHA anabolism pathway, the bacterial isolates were also benzoate-degrading, which may hint at an alternative bioconversion pathway. Further investigations are needed in order to elucidate the mechanism underlying the bioconversion properties of these isolates.

Differences in growth and cellular PHA accumulation were observed among the various groups of bacterial isolates (Table 1). Low growth and PHA contents were observed for Group 1 (0.35±0.07 g L^−1^; 0.05±0.00% CDM) and Group 4 (0.26±0.04 g L^−1^; 0.08±0.01% CDM). These groups clustered closely with the styrene-degraders *Pseudomonas* sp. LQ26 and *Pseudomonas* sp. VLB120, and atrazine-degrader *Pseudomonas* sp. ADP (16, 21, 30) (Fig. 1A). However, the bioaccumulation of PHA has not been previously described in these Pseudomonads. Although these results provided the first evidence of mcl-PHA production in these Pseudomonads, the PHA contents were too low to be considered attractive for application purposes.

Group 3 showed higher growth and PHA content (0.37±0.19 g L^−1^; 6.23±1.46% CDM), and shared a closer evolutionary relationship to existing mcl-PHA producing *Pseudomonas* strains (Fig. 1A). This included *P. fulva* TY16 and *P. putida* F1, which reportedly produced mcl-PHA from benzene, toluene, and ethylbenzene (26, 27); *P. putida* mt-2, which utilizes toluene, *p*-xylene, and various non-aromatic carbons for the production of mcl-PHA, and *P. putida* KT2440, which produces mcl-PHA from glucose and alkanoic acids (40). Group 3 was also clustered closely with *Pseudomonas* sp. TN301 (99% identity with 97% coverage), which could produce mcl-PHA from styrene (25). However, the growth, PHA content, and mcl-PHA mass fraction of Group 3 were approximately 2-folds higher than those of *Pseudomonas* sp. TN301, implying that Group 3 isolates were more efficient at utilizing styrene for the formation of PHA. Mcl-PHA contents can typically reach between 20 and 30% CDM with aromatic carbon substrates (40), which indicated that the PHA content of Group 3 was low. Nevertheless, the close evolutionary neighbors of Group 3, *Pseudomonas* sp. TN301 and *P. putida* F1, reportedly utilized various aromatic compounds for the synthesis of PHA and accumulated mcl-PHA up to 23% CDM and 22% CDM using naphthalene and toluene, respectively (25, 27). This finding suggests that Group 3 bacterial isolates may be able to metabolize other toxic aromatic pollutants and accumulate higher amounts of mcl-PHA. Additional studies will be necessary for further verification.

Among the four groups tested, Group 2 had the highest growth and PHA content (0.63±0.14 g L^−1^; 13.67±5.22% CDM) (Table 1). An isolate, subsequently identified and designated as *P. putida* NBUS12 (Fig. 2A), showed the most growth (0.80±0.03 g L^−1^) and PHA accumulation (23.10±3.25% CDM) on styrene. The cellular PHA content of *P. putida* NBUS12 was similar to that of *P. putida* CA-3 (45) and approximately 9 to 20% higher than other styrene-degrading *Pseudomonas* strains (*i.e.*, *P. putida* CA-1, *P. putida* S12, and *Pseudomonas* sp. TN301) (25, 42), making it one of the highest PHA-producing strains among existing styrene-degrading bacteria and warranted its selection for further investigation.

Genetic analyses revealed *P. putida* NBUS12 as an evolutionarily distinct strain from known styrene-degrading and PHA-producing *Pseudomonas* strains. The 16S rDNA and *phaZ* genes of *P. putida* NBUS12 did not share high similarity with that of *Pseudomonas* sp. TN301 (16S rDNA, 99% identity with 97% coverage) or *P. putida* CA-3 (*phaZ*, 88% identity with 100% coverage) (Fig. 1A and Fig. 1B). Higher genetic similarity was observed with the non-PHA-producing *Pseudomonas* strains *P. putida* 31920-1 (16S rDNA, 100% identity with 99% coverage) and *Pseudomonas* sp. VLB120 (*phaZ*, 100% identity with 100% coverage). While most of the genetic variations observed between the *phaZ* sequences from *P. putida* NBUS12 and *P. putida* CA-3 were attributed to codon degeneracy (PhaZ, 98% identity with 100% coverage) (Fig. S1), some genetic variations resulted in amino acid substitutions (data not shown). Additionally, ClustalW analyses of *phaZ* sequences and PhaZ sequences showed *P. putida* NBUS12 as an evolutionarily distinct branch from *P. putida* CA-3 and other known PHA-producing Pseudomonads (36). This finding suggests that despite its phenotypic similarity to existing bacterial strains, *P. putida* NBUS12 may be a new styrene-degrading and PHA-producing bacterial strain.

A 500-mL volume of cultivation using a higher inoculum amount of *P. putida* NBUS12 led to an improvement in final biomass production (1.28±0.10 g L^−1^) and PHA production (0.42±0.04 g L^−1^), which were 1.6-fold and 2.2-fold higher, respectively, than the shake-flask culture (Table 1). A higher PHA content (32.49±2.40% CDM) and PHA mass fraction (324.90±23.98 mg gCDM^−1^) were also observed and found to be similar to values obtained by *P. putida* CA-3 (28). The extracted PHA polymer mainly comprised C_6_ , C_8_ , C_10_ , C_12_ , C_14_ at a ratio of 2:42:1257:17:1, showing an increased dominance of the C_10_ monomer and an additional C_14_ monomer (Fig. 2B and Fig. 3), which were previously not observed under shake-flask cultivation (Table 1). Variations in monomer content for the same bacterium have been reported previously (34), and may be ascribed to cultivation conditions, which, in turn, influence the activity of enzymes involved in the *de novo* fatty acid synthesis pathway, leading to an increase in the synthesis of the C_10_ monomer and C_14_ monomers (49). PHA monomers such as C_6_ , C_8_ , C_10_ , C_12_ , and C_12:1_ are known to arise from styrene metabolism (25, 27, 42, 45). While the C_14_ monomer has previously been reported as a minor monomer in mcl-PHA formed from glucose, long-chain alkanoic acids, and tallow (34), it currently remains unknown if this monomer could be derived from styrene. We herein demonstrated, for the first time, C_14_ monomer synthesis from styrene.

The properties of the PHA polymer produced by *P. putida* NBUS12 were found to be consistent with those expected for mcl-PHA, and the polymer was likely to resemble thermoplastic elastomers (Table 3) (24, 34). Unlike short-chain-length PHA (scl-PHA), which typically has markedly higher *T_m_* (up to 180°C) and crystallinity (up to 78%) (32), the combination of reduced *T_m_* (50.67°C) and crystallinity (13.57%) made mcl-PHA more malleable and biodegradable (34). Greater biocompatibility over scl-PHA was also reported for mcl-PHA due to the reduced cytotoxicity observed for PHA monomers with longer carbon numbers (37). These attributes collectively meant that mcl-PHA, produced from *P. putida* NBUS12, potentially has high-value medical applications such as soft tissue regeneration, controlled drug delivery matrices, or as plastic coatings and pressure-sensitive adhesives (20, 34). This study’s polymer had higher *M_w_* (101,500 Da) and *M_n_* values (49,300 Da) and a lower PDI value (2.06) than those of the mcl-PHA polymer produced from *P. putida* CA-3 using styrene (45). Higher *M_w_* values are generally associated with greater PHA polymer tensile strength (33, 43), suggesting that the mcl-PHA obtained in this study is more suitable for applications requiring higher tensile strength. The wider temperature range between *T_g_* (−46.05°C) and *T_m_* (50.67°C) and higher DT value (277°C) also indicated that this polymer has a wider processing and application temperature, which may expand its applicability. Nevertheless, the *M_w_* of this study’s polymer was still lower than that of mcl-PHA produced from other carbon sources (*e.g.*, sugars, alkanes, alkenes, and long-chain fatty acids) where *M_w_* values ranged from 124,000 Da to 339,000 Da (34). Factors such as the state of inoculums, cultivation media composition, fermentation conditions, and PHA extraction process have been asserted to influence the *M_w_* of PHA (34). An investigation of the aforementioned factors on the *M_w_* of mcl-PHA from *P. putida* NBUS12 will be the subject of future studies.

## Conclusion

This study demonstrated a simple enrichment strategy for the isolation of bacteria capable of bioconverting the organic eco-pollutant styrene into mcl-PHA from both petrochemical-contaminated and non-contaminated inoculum sources. Twelve styrene-degrading and mcl-PHA-producing Pseudomonad isolates were obtained. The best-performing isolate was the novel strain, *P. putida* NBUS12, which could achieve growth of 1.28±0.10 g L^−1^ and mcl-PHA content of 32.49±2.40% CDM, making it one of the highest PHA-producing strains among known styrene-degrading bacteria. The mcl-PHA polymer from *P. putida* NBUS12 showed monomeric components distinctive from existing phenotypically similar bacterial strains and provided the first evidence of C_14_ monomer synthesis from styrene. Besides saturated monomers (*i.e.*, C_6_ , C_8_ , C_10_ , C_12_ , and C_14_ ), PHA also contained unsaturated monomers, which hold the possibility for further chemical modifications. Chemical modifications greatly impact on the properties of PHA, and expand its usage in medical and environmental fields. The higher degree of monomer heterogeneity may influence and result in different polymer properties, expanding the possible applications of the mcl- PHA polymer derived from a styrene-based source. Collectively, this study successfully increased the limited pool of unique bacterial cultures that may aid in the development of biotechnologies for simultaneous styrene effluent treatment and mcl-PHA recovery. Future studies will focus on improving the production and polymer properties of PHA by *P. putida* NBUS12 in order to enhance the economic viability of this bioprocess.

## Supplementary Information



## Figures and Tables

**Table 1:** Growth and PHA accumulation by bacterial isolates using styrene as the sole carbon source

Group	Bacterial isolates	CDM ±s.d. (g L^−1^)	PHA content ±s.d. (% CDM)	Mass fraction of PHA ±s.d. (mg gCDM^−1^)	PHA monomeric composition C_6_ :C_8_ :C_10_ :C_12_ (wt %)
1	NBUS9 NBIWW1 NBIWW3	0.35±0.07	0.05±0.00	0.55±0.03	0:0:82:18
2	NBUS5 NBUS12 NBIWW6	0.63±0.14 (0.80±0.03)	13.67±5.22 (23.10±3.25)	136.70±52.16 (231.03±32.53)	1:20:76:3 (1:15:82:2)
3	NBUS1 NBUS8	0.37±0.19	6.23±1.46	62.25±14.62	4:30:64:2
4	NBUS7 NBUS11 NBIWW4 NBIWW7	0.26±0.04	0.08±0.01	0.79±0.13	0:0:52:48

Values for *Pseudomonas* isolate NBUS12 are provided within parentheses. Cell yield was obtained by taking the average CDM for each group; Average PHA content was calculated by dividing the PHA yield (g L^−1^) by cell yield (g L^−1^). Results were the mean values of at least three independent experiments (*n*≥3) and s.d. refers to standard deviation.

**Table 2:** Positive growth of *P. putida* NBUS12 on carbon substrates and antibiotics found in Biolog GEN III MicroPlate™

Chemical guild	Substrate	Chemical guild	Substrate
Sugars	*α*-*D*-Glucose	Hexose acids	*D*-Galacturonic acid
	*D*-Mannose		*D*-Gluconic acid
	*D*-Fructose		*D*-Glucuronic acid
	*D*-Galactose		Glucuronamide
	*D*-Fucose		Mucic acid
	*L*-Fucose		Quinic acid
	*L*-Rhamnose		*D*-Saccharic acid
Hexose-phosphate	*D*-Fructose-6-phosphate	Carboxylic acids, esters and fatty acids	*L*-Lactic acid
			Critic acid
Amino acids	*L*-Alanine		*α*-Ketoglutaric acid
	*L*-Arginine		*L*-Malic acid
	*L*-Aspartic acid		*γ*-Aminobutyric acid
	*L*-Glutamic acid		*β*-Hydroxy-*D*,*L*-butyric acid
	*L*-Histidine		Propionic acid
	*L*-Pyroglutamic acid		Acetic acid
	*L*-Serine		
	*D*-Serine	Alcohol	Glycerol
		Antibiotic	Minocycline

**Table 3:** Properties of the PHA polymer produced from styrene by *P. putida* NBUS12

*M_w_* (Da)	*M*_n_ (Da)	PDI	*T_g_* (°C)	*T_m_* (°C)	DT (°C)	Crystallinity (%)
101,500	49,300	2.06	−46.05	50.67	277.00	13.57
