# Supplementary material for: Bioconversion of Styrene to Poly(hydroxyalkanoate) (PHA) by the New Bacterial Strain Pseudomonas putida NBUS12
Source: Microbes Environ. 2015 Feb 14;30(1):76–85. doi: 10.1264/jsme2.ME14138 (PMC4356467; doi:10.1264/jsme2.ME14138)
Supplement: Supplementary file 1 [file 30_76_s1.pdf]

**Table S1.** NMR chemical shift assignments of PHA monomers in mcl-PHA produced from styrene by *P. putida* NBUS12

| | Position[a] | $C_6$ | $C_8$ | $C_{10}$ | $C_{12}$ | $C_{14}$ |
|---|---|---|---|---|---|---|
| $^1$H-NMR | a | 2.52 | 2.52 | 2.52 | 2.52 | 2.52 |
| | b | 5.17 | 5.17 | 5.17 | 5.17 | 5.17 |
| | c | 1.58 | 1.58 | 1.58 | 1.58 | 1.58 |
| | d-1 | 1.25 | 1.25 | 1.25 | 1.25 | 1.25 |
| | d-2 | | 1.25 | 1.25 | 1.25 | 1.25 |
| | d-3 | | 1.25 | 1.25 | 1.25 | 1.25 |
| | d-4 | | | 1.25 | 1.25 | 1.25 |
| | d-5 | | | 1.25 | 1.25 | 1.25 |
| | d-6 | | | | 1.25 | 1.25 |
| | d-7 | | | | 1.25 | 1.25 |
| | d-8 | | | | | 1.25 |
| | d-9 | | | | | 1.25 |
| | e | 0.87 | 0.87 | 0.87 | 0.87 | 0.87 |
| $^{13}$C-NMR | 1 | 169.73 | 169.73 | 169.73 | 169.73 | 169.73 |
| | 2 | 39.44 | 39.44 | 39.44 | 39.44 | 39.44 |
| | 3 | 71.19 | 71.37 | 71.37 | 71.37 | 71.37 |
| | 4 | 36.22 | 34.15 | 34.15 | 34.15 | 34.15 |
| | 5-1 | 18.65 | 25.05 | 25.41 | 25.41 | 25.41 |
| | 5-2 | | 31.86 | 29.52 | 29.52 | 29.52 |
| | 5-3 | | 22.69 | 32.12 | 29.69 | 29.69 |
| | 5-4 | | | 23.03 | 29.52 | 29.69 |
| | 5-5 | | | 25.41 | 29.33 | 29.69 |
| | 5-6 | | | | 32.12 | 29.52 |
| | 5-7 | | | | 23.03 | 29.33 |
| | 5-8 | | | | | 32.12 |
| | 5-9 | | | | | 23.03 |
| | 6 | 14.11 | 14.27 | 14.27 | 14.27 | 14.27 |

[a] Refer to Fig. 3 for atom numbers

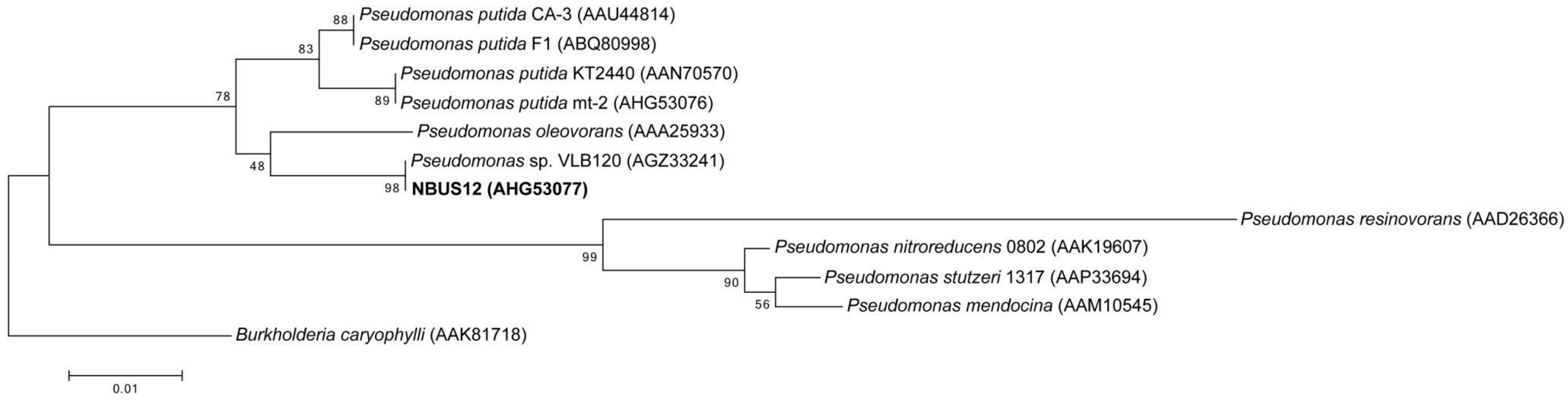


**Fig. S1.** Phylogenetic tree of PhaZ protein sequence from *P. putida* NBUS12 (designated in bold), *Pseudomonas* sp. VLB120 and known PHA-producing Pseudomonad strains (described by Solaiman and Ashby [34]) with *Burkholderia caryophylli* as the outgroup. Genbank accession numbers are provided within parentheses. The tree was constructed using the Neighbor-joining (*p*-distance) algorithm with MEGA5.2 software. The numbers beside the nodes indicated bootstrap values based on 1000 replications. The scale bar represents the estimated number of nucleotide changes per sequence position.
